# Ultrasensitive detection of HIV-1 p24 antigen by a hybrid nanomechanical-optoplasmonic platform with potential for detecting HIV-1 at first week after infection

**DOI:** 10.1371/journal.pone.0171899

**Published:** 2017-02-15

**Authors:** Priscila M. Kosaka, Valerio Pini, Montserrat Calleja, Javier Tamayo

**Affiliations:** IMM–Instituto de Microelectrónica de Madrid (CNM-CSIC), Isaac Newton, 8, PTM, Tres Cantos, Madrid, Spain; Waseda University, JAPAN

## Abstract

Early detection of HIV infection is the best way to prevent spread of the disease and to improve the efficiency of the antiretroviral therapy. Nucleic acid amplification tests (NAAT) have become the gold-standard for detecting low-concentrations of the virus in blood. However, these methods are technically demanding and cost-prohibitive in developing countries. Immunoassays are more affordable and can be more easily adapted for point-of-care diagnosis. However, the sensitivity so far of these methods has been too low. We here report the development of a sandwich immunoassay that combines nanomechanical and optoplasmonic transduction methods for detecting the HIV-1 capsid antigen p24 in human serum. The immunoreactions take place on the surface of a compliant microcantilever where gold nanoparticles are used as both mechanical and plasmonic labels. The microcantilever acts as both a mechanical resonator and an optical cavity for the transduction of the mechanical and plasmonic signals. The limit of detection of the immunoassay is 10^−17^ g/mL that is equivalent to one virion in 10 mL of plasma. This is 5 orders of magnitude better than last generation of approved immunoassays and 2 orders of magnitude better than NAAT. This technology meets the demands to be produced *en masse* at low cost and the capability for miniaturization to be used at the point-of-care.

## Introduction

Acute human immunodeficiency virus infection (AHI) can be defined as the time from HIV acquisition until seroconversion, i.e., the appearance of detectable antibodies to HIV in the blood [1,2]. The duration of this stage is of about four weeks. Detection of AHI is crucial for improvement of the individual’s health [3]. Progressive changes occur after HIV acquisition such as irreversible depletion of CD4 lymphocytes in the gut, replication in the central nervous system, and the establishment of latent HIV reservoirs, which up to date has rendered incurable [4]. Initiation of antiretroviral therapy (ART) during AHI improves host immune control of viral replication and has been associated with improvements in CD4 cell counts over time, reduced systemic inflammation, preserved cognitive function, and a reduced latent reservoir [5]. Detection of AHI is also critical for prevention of HIV transmission. Subjects with acute infection are maximally contagious, because HIV replicates without being checked by the immune system and the amount of virus in blood and genital secretions rises rapidly. In addition, the infectivity potential of the virus during the early infection stage is much higher than in later stages.

The AHI can be diagnosed by detecting in blood either the viral RNA by nucleic acid amplification tests (NAATs) or the HIV capsid antigen p24 by the fourth-generation immunoassays [3,4]. The first method exhibits a detection limit of 20–35 RNA copies/mL, i.e. 10–18 virions/mL, a concentration that typically occurs ~2 weeks after HIV acquisition. Fourth-generation immunoasssays achieve a p24 detection limit of ≈10 pg/mL, a concentration approximately reached between three and four weeks after infection [6]. Although NAATs are very sensitive, these technologies are technically complex and expensive. Immunoassays for p24 detection are simpler and cheaper and have the potential for implementation in low-resource settings where the prevalence of HIV infection is very high. Moreover, HIV-1 p24 antigen should be a more sensitive indicator of the virus presence as a virion approximately contains 2,000–3,000 p24 antigens *vs* only 2 RNA copies [7,8].

Immunoassays for p24 detection are based on enzyme-linked immunosorbent assays (ELISA). Briefly, the targeted antigen is bound to a surface by specific capture antibodies, and subsequently the captured antigen is specifically linked to a primary antibody. Enzyme-linked secondary antibodies are then used to bind a region of the primary antibody. In the final step, an enzyme substrate is added to produce a change of color of the solution that is used as the detection signal. Numerous efforts have been made to lower the limit of detection of p24, mostly focused on improving the signal generation mechanism. In 2012 the best detection limit was obtained by Rica & Stevens using enzymes that control the growth of gold nanoparticles that generate coloured solutions achieving a striking detection limit of ≈10^−6^ pg/mL [9]. However, these assays are complex, involving several steps in the signal amplification. Recently, the generation of an amplified fluorescent signal by nuclease-linked fluorescence oligonucleotide assay has provided a detection limit ≈1–2 pg/mL[10] and the combination of ELISA with thio-NAD cycling that change the light absorbance at 405 nm has demonstrated a detection limit of ≈0.1 pg/mL [11]. More recently, the company Quanterix Corp. obtained a detection limit of ≈3 10^−3^ pg/mL by using digital bead-based ELISA with fluorescent signal [8].

We here develop a sandwich immunoassay based on nanomechanical and optomechanical transduction for detecting p24 antigen in human serum samples that only involves two immunoreaction steps [12]. The technique achieves a detection limit of ≈10^−5^ pg/mL. The technique could dramatically shorten the window period of HIV infection, in which the disease is undetectable, to just a single week.

## Results

### Nanomechanical and optoplasmonic transduction methods

**Fig 1:**
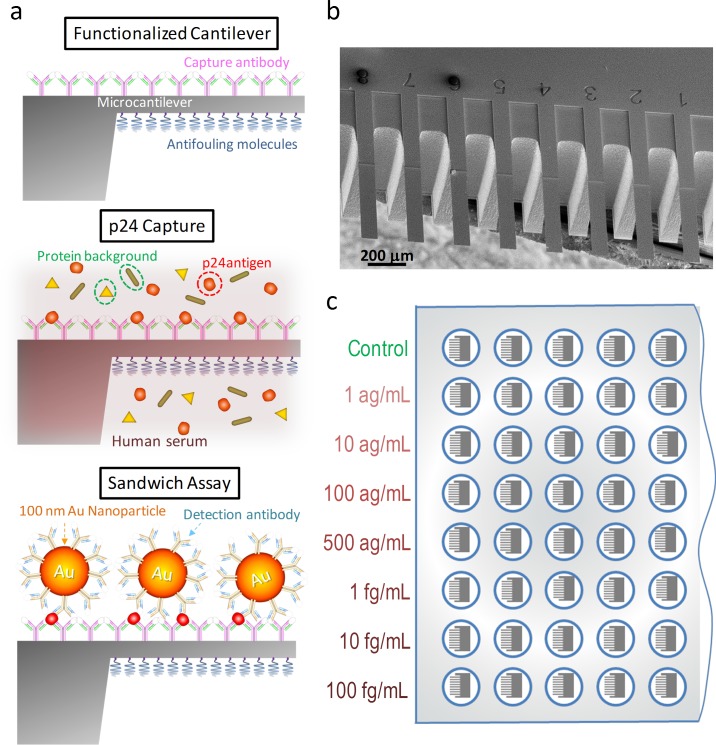
Schematic representation of the p24 sandwich immunoassay. (a) The top surface of the cantilever is functionalized with capture antibodies against HIV-1 p24 antigen (top schematic). Antifouling molecules are immobilized on the bottom surface of the cantilever and voids between the antibodies to minimize nonspecific interactions. The cantilever is then immersed in the human serum sample to allow specific binding of p24 to the cantilever surface (middle schematic). Finally, the p24 antigen captured on the cantilever is specifically linked to 100-nm-diameter gold nanoparticles that carry detection antibodies. (b) Scanning electron microscopy image of the silicon microcantilever arrays used in this work. (c). Schematics of the 96-well microtiter plate format, in which the immunoassays were carried out.

A schematic of the sandwich immunoassay is shown in Fig 1A. Single crystal silicon microcantilever arrays and 100-nm-diameter gold nanoparticles are respectively biofunctionalized with *capture antibodies* and *detection antibodies* by a procedure that ensures optimal recognition efficiency and ultralow fouling capability[12,13] (see Materials and Methods). The microcantilevers are 500 μm long, 90 μm wide and 1 μm thick (Fig 1B). First, the functionalized microcantilever array is incubated in 1 mL of the human serum sample for one hour at 37°C to allow specific binding of the HIV-1 p24 antigens to the *capture antibodies* immobilized on the cantilever surface. Second, after stringent rising of the microcantilever array to remove nonspecific adsorption, the cantilever is incubated with 1 mL of 10 μg/mL solution of the gold nanoparticles at 37°C for 15 minutes for labeling of the captured p24 proteins on the cantilever. Finally, the cantilever is subjected to stringent rinsing to remove the nanoparticles nonspecifically bound. In order to obtain statistically meaningful results and adapt our technique to the medical diagnostic testing, the immunoassay steps were carried out in a 96-well microtiter plate format (Fig 1C). Each functionalized microcantilever array was transferred to a well of the microtiter plate for testing p24 concentrations in human serum from 0 to 0.1 pg/mL. Each array has eight microcantilever sensors, and each concentration was tested by 3–5 microcantilever arrays. The total time of the assay is 4 hours and 45 minutes. The clinical results of an individual could in this way be obtained in the same day with this technique.

**Fig 2:**
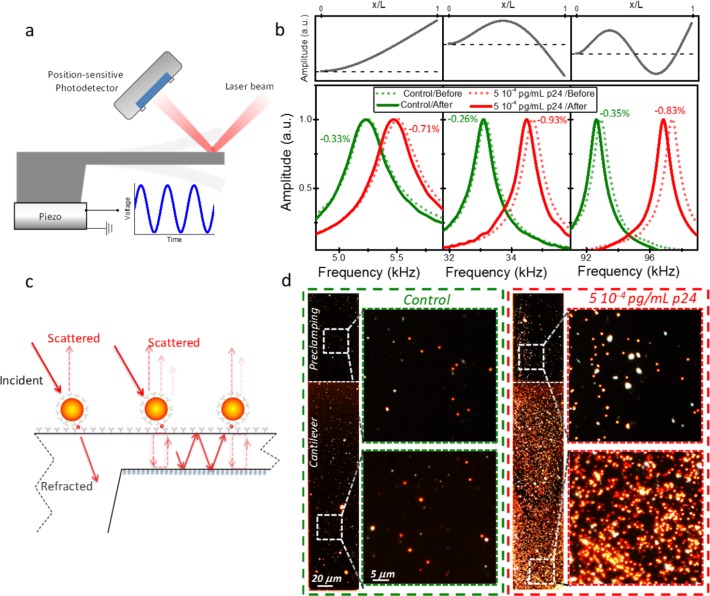
Nanomechanical and optoplasmonic transduction of the immunoreaction product. (a) Schematics of the optical beam deflection method for measuring the microcantilever vibration. A laser beam is focused onto the cantilever free end region. The deflection of the reflected beam due to the cantilever vibration is measured by a position-sensitive photodetector. The microcantilever is driven by a piezoelectric actuator beneath the microcantilever array chip. (b) Resonance frequency peaks (bottom graphs) of the first three vibration modes of a silicon cantilever before (dotted lines) and after (solid lines) the immunoreaction assay for a blank human serum sample (control) and for 5 10^−4^ pg/mL of p24 in human serum. Top panels represent the vibration mode shapes. The relative variations of the resonance frequencies are shown beside the resonance frequency peaks. (c) Schematics of the multiple pathways for light scattering by the gold nanoparticles bound to the microcantilever. (d) Darkfield optical images of the microcantilevers analyzed in (b) after the immunoassays. The images show the microcantilever and chip preclamping regions. At the right zoomed images of regions at the preclamping and the microcantilever are also shown.

Gold nanoparticles act as mass and plasmonic labels; the two signatures are detected by means of a silicon cantilever that serves as mechanical resonator for 'weighing' the mass of the captured nanoparticles and as an optical cavity that boosts the plasmonic signal from the nanoparticles. In the first method (nanomechanical transduction), we measure the microcantilever vibration by the scanning optical laser beam deflection technique [14,15] (Fig 2A). The microcantilever array is driven by a piezoelectric actuator located beneath the chip base. We here measure the mechanical resonance frequencies of the microcantilevers corresponding to the first three flexural vibration modes. These measurements are carried out in air, before the microcantilever functionalization and after completing the immunoassay. In this way, the biosensing surface of the microcantilevers is never dried and heated by the laser during the immunoassay process, which can lead to denaturation of the proteins and loss of biofunctionality. Assuming that the added mass is uniformly distributed over the microcantilever surface, the relative variation of the resonance frequencies is given by[16],

$$\frac{\Delta f_{n}}{f_{n}}=-\frac{1}{2}\frac{\Delta m}{m}$$ (1)

where *f*_*n*_ is the resonance frequency of the *n*^*th*^ vibration mode in air prior to the functionalization, Δ*f*_*n*_ is the shift of the resonance frequency after the immunoassay, *m* is the cantilever mass and Δ*m* is the increase of the cantilever mass due to the immunoassay steps. The measurement of several vibration modes reduces the error and minimize the effect of adsorbed contaminants that exhibit non uniform spatial distribution over the microcantilever surface, giving uncorrelated frequency shifts as opposed to uniform adsorption. Fig 2B shows the resonance frequency curves of the first three vibration modes before and after the immunoassay for a control sample (human serum without p24) and for 5 10^−4^ pg/mL of p24 in human serum. The shapes of the vibration modes are also shown. The positive sample provided changes of the resonance frequency of about -0.8%, whereas these changes were reduced to -0.3% with the control samples. The signal background mainly comes from the mass added by the functionalization of the microcantilever.

The second method for detecting the gold nanoparticle labels is optoplasmonic transduction [12]. The 100 nm diameter gold nanoparticles exhibit a localized plasmon resonance centered at a wavelength of 620 nm that result in a resonant enhancement of the light scattering at near wavelengths. The microcantilever is also an optical cavity in which the light can be confined between the two opposite surfaces of the microcantilevers for certain wavelengths. When nanoparticles are bounded to the microcantilever, the optical cavity modes and the localized surface plasmon modes couple each other at very characteristic frequencies and form hybrid plasmonic supermodes that enhance the scattering of the nanoparticles [12,17]. A schematic of the enhanced scattering in this structure is depicted in Fig 2C. When the light interacts with a nanoparticle on the preclamping region, the scattered light is only collected in a solid angle given by the numerical aperture of the objective, referred to as backward scattering. When the nanoparticle is on the microcantilever, in addition to the backward scattering, multiple pathways assist to enhance the scattering by a single nanoparticle. One pathway involves the amplification of the forward scattering of a nanoparticle by multiple internal reflections. In a second pathway, the refracted light undergoes multiple internal reflections in the optical microcantilever cavity, resulting into a cascade of scattering interactions at the neighboring nanoparticle sites. Fig 2D shows the darkfield images of the two microcantilevers previously analyzed by the nanomechanical transduction method (Fig 2B). We observe two effects: i) the scattering intensity of the microcantilever exposed to human serum with 5 10^−4^ pg/mL of p24 is much higher than in the control experiment (human serum without p24); ii) in the case of the p24 detection assay, the scattering intensity is weak in the preclamping region, whereas it is largely enhanced in the microcantilever surface due to the coupling between the dipolar plasmon resonance of the nanoparticle and the optical cantilever cavity resonances.

### Detection of p24 in human serum

**Fig 3:**
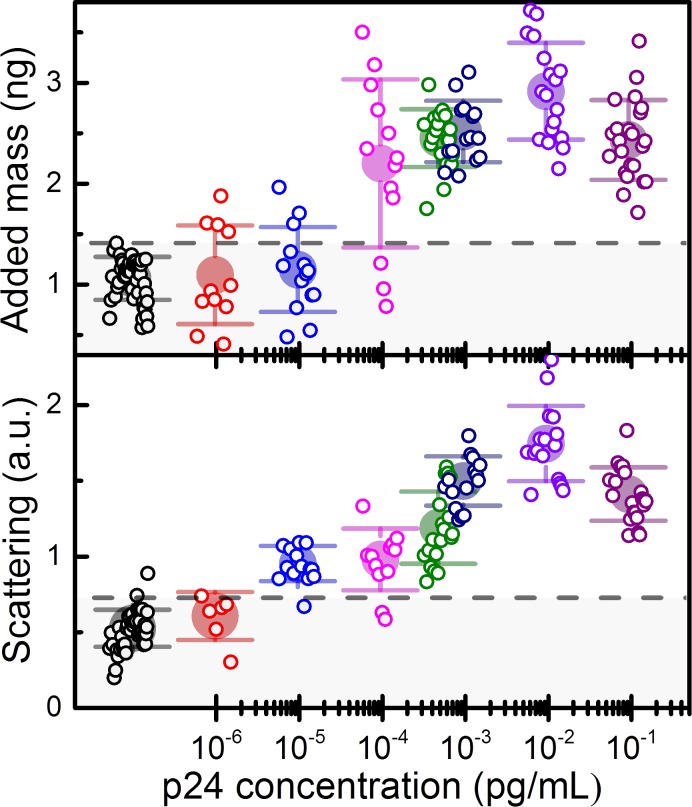
Nanomechanical and optoplasmonic detection of p24 in human serum. Top graph: added mass inferred from the changes in the frequency response of the first three vibration modes of the microcantilevers. Bottom graph: scattering signal obtained from processing the darkfield images of the microcantilever after the immunoassay. The small symbols represent results of single microcantilevers. Big symbols and error bars represent the mean and error of all results at each concentration. Dashed line represents the limit of blank as defined in the text.

Fig 3 summarizes the nanomechanical (added mass) and optoplasmonic signals (scattering intensity) as a function of p24 concentration in human serum. In order to calculate the added mass, we first calibrated the mass of each of the microcantilevers used in the assays (Eq (1)) by using the well-known equation $m=\frac{k_{n}}{{\left(2\pi f_{n}\right)}^{2}}$, where *k*_*n*_ is the dynamic spring constant of the n^th^ vibration mode that depends on the cantilever dimensions and Young’s modulus[18]. Since the dimensions and mechanical properties of the cantilever can present uncertainties, we determined the spring constant from the experimental frequency response of the cantilever by the Sader’s method [19,20]. The calibration provided a mean and standard deviation of the unloaded cantilevers of 104.2±8.4 ng. The added mass is calculated for each vibration mode and the average value is used as the nanomechanical signal of the assay. The control assays (N = 46) provided an added mass of 1.04±0.22 ng that is approximately the estimated mass for the functionalization layers (antibodies and PEG layer) (Fig 3, top). The p24 starts to be clearly detected for concentrations above 10^−4^ pg/mL and the added mass quickly saturates for concentrations higher than 10^−3^ pg/mL. The saturation mass corresponds to a density of 3–4 nanoparticles per μm^2^.

The optoplasmonic signature of the Au nanoparticles is detected by imaging the microcantilevers by ordinary optical dark field microscopy (example of such images are shown in Fig 2D). The red-channel of the scattering intensity is integrated over the microcantilever surface, and the resulting value is normalized to the result obtained in a clean silicon surface. Bottom graph of Fig 3 shows the resulting normalized scattering signal as a function of the p24 concentration in human serum. In this transduction mode, the lowest concentration that can be clearly distinguished from the control is 10^−5^ pg/mL, one order of magnitude lower than in the nanomechanical transduction mode. The scattering intensity signal saturates for p24 concentrations above 10^−3^ pg/mL as it occurs with the mechanical transduction method.

The improved limit of detection of the optoplasmonic transduction method is attributed to its higher specificity to the gold nanoparticles. In the nanomechanical method, although the relative change of the resonance frequency is dominated by the mass of the nanoparticle labels, the mass arising from the functionalization steps, nonspecific adsorption and contamination provides a noise floor that ultimately limits the detection limit. The measurement of several vibration modes allows to decrease the noise floor. In the optoplasmonic transduction method, the scattering signal at wavelengths near red mainly arises from the local surface plasmon resonance of the nanoparticles. In addition, the unavoidable contaminants are masked in the darkfield images by developed filters that operate in the relevant size and wavelength domains. All these factors contribute to a better detection limit. It is important to emphasize that having several transduction methods in the same platform largely increases the reliability and robustness of the assay [12], which is critical in clinical diagnosis and for monitoring of donor plasma.

## Discussion

**Fig 4:**
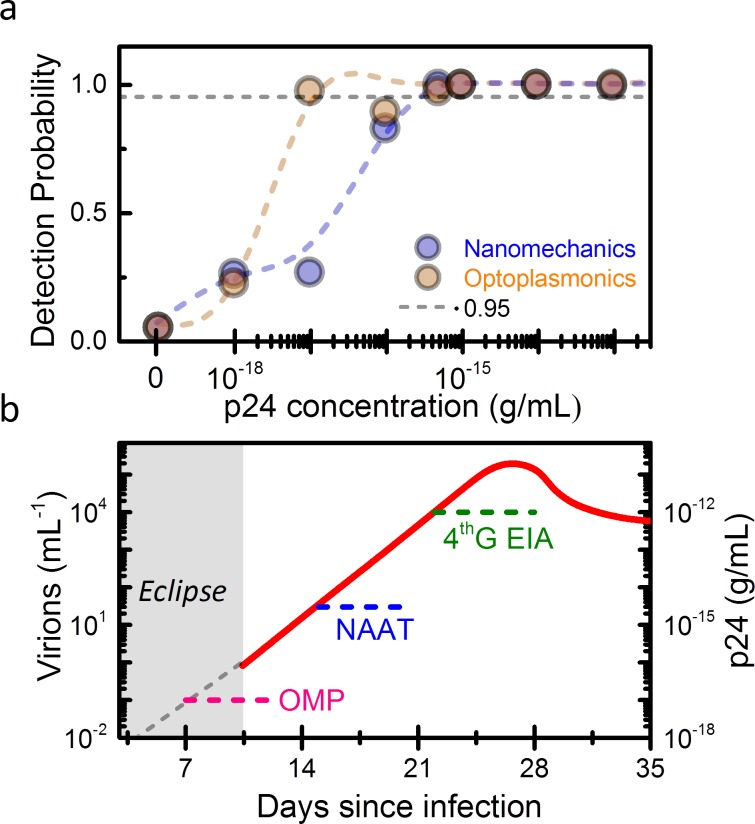
(a) Probability of the nanomechanical and optoplasmonic signals being above the Limit of Blank as a function of p24 antigen concentration in human serum. The grey dashed line represents the 95% probability that determines the Limit of Detection. (b) Estimation of viral load in blood during the first five weeks after HIV infection [1,3]. The limits of detection and the periods of detection of the fourth-generation immunoassays (4^th^ G EIA), nucleic acid amplification testing methods (NAAT) and the presented optomechanoplasmonic method (OMP) are indicated.

We find that the nanomechanical and optoplasmonic signals show a narrow dynamic range, and exhibit a near flat dependence for concentrations above 10^−3^ pg/mL. Although this limits the capability of the assay for quantification, the extremely low detection limit can be harnessed for detecting HIV infection at early stages that would otherwise remain undetectable with the current techniques. We determine the Limit of Detection (LoD) of the presented immunoassay following the guidelines of the Clinical and Laboratory Standards Institute (CLSI)[21]. Firstly, we calculate the Limit of Blank (LoB) defined as the mean signal plus 1.645 times the standard deviation obtained in serum samples devoid of analyte (dashed lines in Fig 3). LoB is used as threshold to determine whether an assay is positive or not. Assuming a Gaussian distribution of the analytical signals, this threshold implies a 5% of false positives. In Fig 4A, the probability of detection as a function of the p24 concentration is plotted for the nanomechanical and optoplasmonic transduction methods. In this calculation, we assume Gaussian distribution for the resulting data. LoD is determined by the lowest concentration that provides results with a probability of 95% of being above the LoB. We find a LoD for the mechanical transduction method of 5 10^−4^ pg/mL. This value is more than 4 orders of magnitude better than that obtained with the fourth-generation immunoassays [6]. The LoD for the optoplasmonic transduction is even better, 10^−5^ pg/mL.

We analyze now the capability of our technique for detecting early HIV infected individuals. In Fig 4B, we represent the viral load and corresponding p24 protein concentration in blood during the first five weeks after infection according to the Fiebig’s staging [1,3]. Approximately, 1 virus per mL is equivalent to 10^−4^ pg/mL of p24. Although the risk of infection depends on the transmission route, the timing of the appearance of viral and host markers of infection is generally uniform and follows an orderly pattern. After infection, there is an initial eclipse phase, in which the HIV-1 is replicating in local and lymphoid tissues and it has not disseminated in the systemic circulation. The eclipse phase is estimated to last 10 days, although the period is defined on basis of the LoD of the most sensitive analytical methods for detecting the virus in blood, so far NAAT methods. Once HIV disseminates to the systemic circulation, HIV quickly replicates to a peak level with a doubling time of 20.5 h. NAAT can detect 60 HIV RNA copies/mL (each virus contains two RNA copies), which implies the detection of HIV infection approximately 2 weeks after infection. Currently, NAAT can only be performed in well-resourced laboratories and it still suffers from long measurement times and high complexity [4]. The p24 antigen can be detected by fourth-generation immunoassays that have been adopted by most of well-resourced countries as the first-line screening tests since 2014. However, the LoD of these assays is ≈10 pg/mL, only allowing the detection of HIV 3–4 weeks after infection.

Our immunoassay can achieve a LoD of 10^−5^ pg/mL that is equivalent to detecting one virion in 10 mL of plasma. This is 5 orders of magnitude better than fourth-generation immunoassays and 2 orders of magnitude better than NAAT. Based on the replication rate of the virus at the ramp-up phase of viremia, it is estimated that the presented immunoassay could detect HIV in blood just one week after infection, that it was considered as eclipse phase so far. The results of the developed p24-test can be reported in less than 5 hours after blood extraction from the patient. The capability of the technique for early detection and reporting is critical in several aspects. First, the acute phase represents a phase of very high infection risk that leads to a disproportionate share of HIV transmissions (in the rhesus macaque model, virions are up to 750 times more infectious than in chronic phase[22]). Detection at this phase can largely contribute to reduce the spread of the disease. Second, early detection of disease allows rapid initiation of antiretroviral treatment with potential for long-term clinical benefits. Third, early detection of HIV infection is still rare in low-resourced countries. A low-cost immunoassay with a sensitivity much higher than NAAT can represent an extraordinary opportunity for drastic reduction of the disease transmission. The presented technology is suitable for miniaturization and low-cost production. Optical and mechanical transducers such as microcantilever arrays and optical cavities are fabricated *en masse* by well-established semiconductor technology [16]. Similarly, optical components such as lasers and photodiodes can be acquired at very low cost [23]. In addition, smartphones now can be transformed in powerful and economic optical microscopes to be used in the field setting [24].

## Conclusions

We here report the development of a sandwich immunoassay that combines nanomechanical and optoplasmonic transduction methods for detecting HIV-1 capsid antigen p24 in human serum. The limit of detection of the immunoassay is 10^−17^ g/mL that is equivalent to one virion in 10 mL of plasma. This is 5 orders of magnitude better than last generation of approved immunoassays and 2 orders of magnitude better than NAT methods. This LoD reduces the undetectable phase after infection to just one week. The technology meets potential to be produced *en masse* at low cost and capability for miniaturization to be used at point-of-care.

## Material and methods

### Materials

Sulphuric acid (ACS reagent, 95–98%), human serum from human male AB plasma (USA origin, sterile filtered, ref. 4522, Lot# SLBJ3904V), hydrogen peroxide (H_2_O_2_ 30%), (3-glycidyloxypropyl)trimethoxysilane (98%), Nα,Nα-Bis(carboxymethyl)-L-lysine hydrate (97%, TLC), dry toluene (99.8%), N-hydroxysulfosuccinimide sodium salt (sulfo-NHS), N-(3-dimethylamino propyl)-N’-ethylcarbodiimide hydrochloride (EDC), (Aminoethyl)polyethylene glycol (5,000 Da), 2-(N-Morpholino)ethanesulfonic acid (MES), bicarbonate buffer solution, phosphate buffered saline (PBS), sodium chloride, sodium phosphate dibasic, sodium phosphate monobasic and Tween® 20 were purchased from Sigma-Aldrich (St. Louis, USA). Spherical 100-nm-diameter gold nanoparticles coated with 5 nm thick carboxyl polymer (C11-100-TC-50) were acquired from Nanopartz (USA). Silicon cantilever arrays were purchased from Micromotive GmbH (Mainz, Germany). The nominal length, width and thickness of the cantilever are 500, 90 and 1 μm, respectively. The immunoassays were carried out with purified monoclonal anti-HIV-1/2 (HIV-018-48304, Capricorn Immunoreagents Perfected, USA), monoclonal Anti-HIV type1 p24 clone 39/5.4A (ZeptoMetrix, USA) and recombinant HIV-1 gag p24 antigen (Virogen, USA). 96-well microtiter plate format from Eppendorf Deepwell Plates.

### Antibody conjugation to gold nanoparticles

The detection antibody, monoclonal Anti-HIV type1 p24 clone 39/5.4A, was immobilized onto the surface of the carboxyl-polymer coated 100-nm-diameter gold nanoparticles (GNP). First, 50 μL of 3.25 mg/mL GNPs in PBS were mixed with 50 μL of 1 mg/mL of the detection antibody in PBS. Then, 500 μL of Milli-Q® water was added to the solution. The resulting solution was vortexed for 1 minute at 25°C (Solution A). Immediately, 50 μL of 1 mg/mL of EDC in Milli-Q® water was added to the Solution A. The mixture was incubated at 25°C for 1 hour and then it was centrifuged at 4°C and 6000 rcf for 10 minutes. The supernatant was removed to 50 μL and the remaining solution resuspended in 650 μL of PBS. This step was repeated twice. The nanoparticle solution was then centrifuged one more time and the pellet formed was mixed with 600 μL of 1 mg/mL BSA in PBS + 0,05% Tween® 20 for 1 hour at 25°C to block the uncoated voids on the gold nanoparticle and avoid non-specific interactions. The solution was then centrifuged at 4°C and 6000 rcf for 10 minutes. The supernatant was removed to 50 μL and the remaining solution was mixed with 650 μL of PBS. This step was repeated twice. The concentration of the conjugated GNPs in solution was measured by a BioSpectrophotometer from Eppendorf (Germany) and it was stored in refrigerator at 4°C until its use.

### Functionalization of microcantilever arrays and immunoreactions

Prior to their functionalization, the microcantilever arrays were cleaned with piranha solution (3 H_2_SO_4_: 1 H_2_O_2_) to remove all the organic residues on the surface *(caution*: *piranha solution is extremely corrosive*, *reactive and potentially explosive)* for 15 minutes at room temperature. The cantilevers were extensively rinsed with Milli-Q® water and dried under a stream of nitrogen. Each cantilever was dipped into a 2 mL 0.2% solution of (3-glycidyloxypropyl)trimethoxysilane in dry toluene overnight at room temperature. The samples were then washed with toluene, Milli-Q® water and dried under N_2_. The cantilevers were then incubated with 600 μL of a 100 mM NTA solution in 50 mM carbonate buffer (pH 9.5) overnight at 25°C. The cantilevers were then rinsed with 50 mM carbonate buffer pH 9.5, Milli-Q water and dried under N_2_. The carboxyl groups at the cantilever surface were activated by immersion in a mixed solution of 100 mM EDC and 150 mM sulfo-NHS both dissolved in 10 mM MES pH 5.5 (600 μL for each cantilever). The cantilevers were incubated for 45 minutes at 37°C. Immediately, the samples were rinsed with 10 mM MES.

Right after the surface activation step, the antibodies were immobilized on the top side of the cantilevers using the drop method described elsewhere [11,12]. The functionalization procedure involves covalent and oriented immobilization of the antibody with a recognition capability higher than 90%. A solution of 50 μg/mL of monoclonal anti-HIV-1/2 (HIV-018-48304), was prepared in 10 mM MES (pH 5.5), the volume of the antibody solution used for each cantilever in this step was 200 μL. The cantilevers were incubated for 2 hours at 37°C. After that, the samples were washed with 10 mM MES (pH 5.5) and rinsed with Milli-Q® water. The samples were incubated with 1mL of 10 mM sodium phosphate buffer (pH 8.0) with 0.3 M NaCl for 45 minutes at 37°C to desorb loosely bound antibodies. The cantilever surface was subsequently blocked to prevent nonspecific adsorptions; the cantilevers were dipped into a 1mL solution of 1 mg/mL (aminoethyl)polyethylene glycol (PEG) solution in 10 mM MES with 0.05% Tween® 20 (pH5.5) overnight at 4°C. Thereafter, the samples were stringent washed with MES pH 5.5 with 0.05% Tween® 20 (pH5.5).

The functionalized cantilevers were incubated for 1 hour at 37°C with 1 mL solutions of HIV-1 p24 antigen in human serum. The p24 concentrations ranged from 0.1 pg/mL to 10^−6^ pg/mL. For the control experiments, the functionalized cantilevers were incubated with 1mL of human serum without p24 antigen. Right after, the cantilevers were washed with MES pH 5.5 + 0.05% Tween® 20 and MES pH 5.5. For the sandwich assay, the cantilevers were dipped into 1 mL of 10 μg/mL solution of spherical gold nanoparticles functionalized with the detection antibody (monoclonal Anti-HIV type1 p24 clone 39/5.4A) prepared in MES pH 5.5 with 0.05% Tween® 20. The samples were incubated at 37°C for 15 minutes, washed for three hours with 1.5 mL of MES pH 5.5 + 0.5% Tween® 20, extensively rinsed with Milli-Q® water and dried under a stream of N_2_.

### Measurement of the nanomechanical response

The vibration of the microcantilever arrays was measured by the scanning laser beam deflection technique described elsewhere [13,14]. The readout technique combines the optical beam deflection method and the automated 2D scanning of the laser beam over the microcantilever array area. We used a 3 mW red laser diode (Schafter-Kirchhoff, 635 nm) and linear voice-coil actuators for the laser scanning (Physik Instrumente GmbH& Co.). The laser spot size is ≈1 μm. The reflected beam is collected in a position-sensitive detector (PSD) designed by MecWins S.L. that provides total light collected on the PSD and the two-dimensional coordinates of the reflected laser spot on the PSD, independently of any light intensity fluctuations. The microcantilever array is driven by a piezoelectric actuator located beneath the chip base. The frequency of piezoelectric actuator signal is swept in the range of the frequency response of the first three vibration modes of the microcantilevers.

### Dark-field microscopy

Dark-field microscopy of the microcantilevers after the immunoassay was carried out in an optical microscope (Nikon Eclipse, Japan) equipped with an Peltier-cooled color CCD camera (DSRi, Nikon—Japan) connected to a PC through a camera controller (DS-U3, Nikon—Japan) and a bright/dark-field objective 50x (N.A. 0.8, LU Plan Fluor, Nikon—Japan). The images were processed with a home-made software developed in Matlab®. The program applies a mask based on size and color to the image for filtering-out the signal that arises from contamination of the surface. The scattering intensity is calculated by integrating the red channel intensity over the unmasked surface of the microcantilever. The scattering intensity signal is normalized to the scattering intensity obtained on a clean silicon surface.
